# The Mediating Role of Emotional Intelligence in the Relationship Between School Principals’ Sustainable Leadership Behaviors and Diversity Management Skills

**DOI:** 10.3389/fpsyg.2021.774388

**Published:** 2021-12-23

**Authors:** Semih Çayak, Menekşe Eskici

**Affiliations:** ^1^Republic of Turkey Ministry of National Education, İstanbul, Turkey; ^2^Curriculum and Instruction Program, Department of Educational Sciences, Faculty of Science and Literature, Kırklareli University, Kırklareli, Turkey

**Keywords:** sustainable leadership, emotional intelligence, diversity management, school principals, mediating role

## Abstract

The purpose of this research is to examine the mediating role of emotional intelligence in the relationship between school principals’ sustainable leadership behaviors and their diversity management skills. For this purpose, this research, which was designed in the relational survey model, was carried out on teachers. The data of the study were collected using the “Sustainable Leadership Scale,” “Diversity Management Scale,” and “Emotional Intelligence Scale.” Descriptive statistics, Pearson product-moment correlation coefficient, *t*-test, one-way ANOVA analyses and structural equation model were used in the analysis of the data. As a result of the research, it was found that the school principals’ sustainable leadership behavior levels, their ability to manage differences and their emotional intelligence levels were high according to teacher perceptions. According to the correlation analysis, it was found that there is a positive and significant relationship between sustainable leadership, diversity management, and emotional intelligence. In addition, path analyses to examine the mediator variable effect revealed that emotional intelligence has a full mediating role in the relationship between school principals’ sustainable leadership behaviors and their diversity management skills. In addition to the research, it was also examined whether teachers perceptions of school principals on sustainable leadership behaviors, diversity management skills, and emotional intelligence levels differ significantly according to teachers’ gender, professional seniority, educational status and the level of education they work in. In addition to this, they have shown that they need to use their emotional intelligence effectively.

## Introduction

Changes in social, political, technological and economic life and globalization affect the philosophies, cultures and strategies of organizations. This change leads to great differentiations especially in the workforce profile (Clegg and Carter, 2009; Almeida and Chase-Dunn, 2018). In this process, the management of human resources, which has become the most basic element of service and production in organizations has gained more importance, so new approaches to management styles and human relations have begun to be needed (Teece, 2018), because every employee in organizations has different characteristics and values. For this reason, knowing the reflections of differences such as demographic and individual characteristics, personality, socio-cultural values, ability-skills and perception, personal conceptualization characteristics, emotional adaptation characteristics, age-experience, gender on the management, individual and organizational benefits and drawbacks of the employees in the organizations will make it possible to evaluate the differences in managerial policies and practices in line with the goals of the organization. Thus, the potential power of each individual making up the organization will be utilized at the maximum level (Campbell, 2000; Lysova et al., 2019; Yahyayeva, 2020).

The concept of managing diversity together gains importance in terms of establishing a better functioning global management system as well as better management of organizations (Pless and Maak, 2004). New administrative structures will rise on the basis of democracy and equality based on respect for the differences. If diversities are not seen as a problem and they can participate in rich management systems brought by diversity, the importance of developing knowledge and skills on the diversity management will increase. At this point, organizational managers have great responsibilities (Trittin and Schoeneborn, 2017). Organizational managers will be able to both provide a competitive advantage against other organizations and maintain their organization for a long time if they can see the diversities of organizational members as a wealth with their leadership behaviors and use them effectively in the context of realizing organizational goals (Coleman, 2012). It is thought that this may be closely related so to the sustainable leadership behaviors of organizational managers (Urbancová et al., 2020; Zeltina, 2020). As a matter of fact, the sustainable leadership approach, which is one of the leadership types on which researches are frequently conducted, is based on an understanding that supports and protects diversities in organizations and argues that a sustainable organizational system can only be realized in this way. Leaders are people who have the power to control, influence and direct the emotions, thoughts and most importantly the behaviors of individuals. From the point of view of educational organizations, school principals will be described as successful leaders when they have the ability to recognize their own personal feelings, be aware of their limits, understand and attach importance to the feelings of others, control stress and anger, be empathetic, and be successful in human relations (Ergin, 2008; Ismayılov, 2019). This seems to be closely related to the emotional intelligence levels of leaders.

## Theoretical Background

### Sustainable Leadership

People live in groups and need a leader to manage the group they belong to and lead them to their goals. This leader, who is at the head of the group, unites the individuals in the group around a goal and increases their courage and energy. In order to achieve this, leaders are expected to have certain characteristics.

Sustainable leadership which aims to enable organizations to build their current situation toward a successful future rather than focusing only on long-term and financial success (Hargreaves, 2007), requires encouraging systematic innovation and providing quality products, services and solutions (Avery and Bergsteiner, 2011). Davies (2007), on the other hand, describes sustainable leadership as “one of the key factors” that underlines the long-term development of the school, and states that sustainable leadership fosters a leadership culture based on the moral purpose of the school, which in turn ensures success for the benefit of all. In this context, passion for continuous improvement, balancing between past tradition and innovations, thinking about the process, encouraging the participation of all members of the organization, developing strategic measures to ensure success and establishing school-society partnership should be characterized by such features (Iliško and Badyanova, 2014).

Sustainable leadership has some benefits for organizations. Some of these benefits, often highlighted in the literature are: Sustainable leadership practices are estimated to improve long-term organizational performance to varying degrees (Suriyankietkaew and Avery, 2016). Sustainable leadership can respond quickly and flexibly to all stakeholders (Avery and Bergsteiner, 2011) and thus, overall stakeholder satisfaction of organizations that adopt sustainable leadership practices increases (Suriyankietkaew and Avery, 2014). Sustainable characteristics of those who manage educational environments also require them to be sensitive to the characteristics of the individuals they work with.

### Diversity Management

The management of diversity is based on accepting individual differences in organizations as they are, not making any discrimination against any person or group, and evaluating the diversities of people in various dimensions in line with individual and organizational purposes. Diversity management is a comprehensive management philosophy that aims to reveal the potentials of all employees in the organization and to evaluate and benefit from these diversities in practice (Jabbour et al., 2011). This philosophy envisages that individuals and groups in the organization protect their own characteristics and that it affects the organization as well as the individual, and that the whole of common values in the organization is formed. Thus, it aims to reveal all the talents of the employees and to develop them in a way that contributes to the goals of the organization (Shen et al., 2009; Urbancová et al., 2016).

As educational organizations, schools contain students and teachers with many different characteristics (Bryan, 2010). Therefore, the diversity management skills of school principals will be of great importance in the effective and efficient management of schools, whose inputs and outputs are recognized, and which contain individuals with so many different characteristics (Young et al., 2010). Thus, it will be possible to reveal all the talents of the employees in the school organization and to develop them in a way that will contribute to the goals of the organization. The process of managing diversities is closely related to the management of emotions. The higher an individual’s emotional intelligence, the higher his or her ability to manage their emotions and thus diversities.

### Emotional Intelligence

Salovey and Mayer (1990), influenced by Gardner’s (1983) multiple intelligence types, discussed that the individual’s ability to perceive and regulate his own and other individuals’ emotions differ and developed the first emotional intelligence model (Gürdere, 2015). Titrek (2013) defined emotional intelligence as understanding the feelings, emotions and thoughts of ourselves and those we communicate with, empathizing with others, managing our emotions by leaving aside fears and thus getting optimum efficiency from our emotions. Goleman (2016), one of the leading researchers in the field of emotional intelligence and the most widely accepted and detailed definition of emotional intelligence in the literature, defines emotional intelligence as; the ability to mobilize oneself, to control impulses, to regulate mood, not to let troubles interfere with thinking, to put oneself in someone else’s shoes, and to hope.

Employees with high emotional intelligence have personality traits that recognize and control their emotions, as well as understand and manage the emotions of others (Dulewicz and Higgs, 2000). For this reason, emotional intelligence gives the individual many advantages in business life and helps the organization to create positive emotions instead of negative emotions and to show more positive behaviors to employees (Gül et al., 2014). In this context, emotional intelligence has a special place in schools as educational organizations, as in other organizations. Because school principals, who know themselves well and can direct their emotions, give more positive reactions to the behaviors of teachers and students. They know what they feel and what emotions they experience, and they can direct the emotions and thoughts of school members in this direction in order to create a better school environment (Weisinger, 1998; Danilov and Mihailova, 2020).

## Conceptual Framework

### The Relationship Between Sustainable Leadership, Diversity Management, and Emotional Intelligence

Within the scope of the research, studies on emotional intelligence, sustainable leadership and the diversity management were examined. It has been observed that these variables have been studied on different samples such as educational institutions and other businesses. However, no research has been found in the literature in which these three variables are used together, both on educational organizations and on other organizations. There are explanations in the literature that sustainable leadership positively supports the management of diversity in organizations (Morrison and Milliken, 2000; Pinder and Harlos, 2001). In addition, one of the principles of sustainable leadership stated by Hargreaves and Fink (2006) is diversity. The principle of diversity refers to allowing all voices to be heard within the organization. On the other hand, Lambert (2011) who conducts research on sustainable leadership, examined the principles of sustainable leadership in two categories as inclusiveness and developmentalism. Among these principles, inclusiveness states that sustainable leadership should respect and support diversities within the organization. Similarly, Lambert (2011) examines the elements of sustainable leadership under six headings: “creating personnel capacity, strategic distribution, unification, generating long-term goals from short-term goals, diversity and protection,” and emphasizes the importance of diversity in sustainable leadership. Therefore, it can be considered as an expected situation that sustainable leadership supports the management of diversity positively.

Emotional elements lie on the basis of the leader’s being understood by the organization. For this reason, it has become necessary for a leader to have emotional intelligence skills to be effective and successful. Goleman (2017) stated that the reason why emotional intelligence is very important in the success of the leader is that this dimension of leadership, which is related to emotions, determines the course of what the leader does. In this respect, although organizational managers emphasize that they have effective sustainable leadership behaviors, some studies have shown that emotional intelligence levels are also effective in realizing these behaviors. E.g., Ulrich and Smallwood (2013), who classified the principles that guide sustainable leadership as “simplicity,” “time,” “accountability,” “resources,” “tracking,” “melioration,” and “emotion,” and drew attention to the importance of “emotion” in sustainable leadership in their last principle. According to Ulrich and Smallwood (2013), sustainable change is also a matter of the heart and needs a strong emotional person rather than an intellectual person.

Based on the information presented above, it is thought that there may be a significant relationship between sustainable leadership and the management of diversity in organizations. In addition, in organizations where people with different characteristics come together, it is possible that the emotional intelligence levels of leaders can play a mediating role between these two variables. In the literature, emotional intelligence may be associated with both sustainable leadership (Badri-Harun et al., 2016; Augusty and Mathew, 2020) and diversity management (Gardenswartz et al., 2010; Kaufmann and Wagner, 2017). However, no study was found in the literature in which these three variables were used together. Based on these considerations, in this study, the mediating role of emotional intelligence in the relationship between sustainable leadership and diversity management was examined. In addition, it was also examined whether the opinions of the participants regarding these three variables showed statistically significant differences according to their demographic characteristics. The research was carried out with teachers working in schools, which are the organizations where human relations are most intense.

### Purpose of the Research

The aim of this research is to examine the relationship between school principals’ sustainable leadership behaviors, diversity management skills and emotional intelligence levels according to teachers’ opinions. In this direction, answers to the following questions were sought in the study.

(1) What are the teachers’ perceptions of the school principals’ sustainable leadership behaviors, diversity management skills and emotional intelligence levels?
(2) Do teachers’ perceptions of school principals’ sustainable leadership behaviors, diversity management skills and emotional intelligence levels show a statistically significant difference according to teachers’ demographic characteristics?
(3) According to teachers’ perceptions, is there a significant relationship between school principals’ sustainable leadership behaviors, diversity management skills and emotional intelligence levels?
(4) According to teachers’ perceptions, does emotional intelligence have a mediating effect on the relationship between school principals’ sustainable leadership behaviors and their diversity management skills?

## Materials and Methods

### Research Model

This research, which examines the relationships between school principals’ sustainable leadership behaviors, diversity management skills, and emotional intelligence levels according to teachers’ perceptions, was designed in the relational survey model, one of the quantitative research methods. In this study, the mediating role of emotional intelligence in the relationship between sustainable leadership and diversity management was tested.

### Population and Sample

The population of the research consists of 11,025 teachers working in Pendik (7,368) and Kartal (3,657) districts of Istanbul in the 2020–2021 academic year. Simple random sampling method was used to determine the sample. It is considered sufficient for the sample that can represent the population, which is between 10,000 and 15,000 with a 5% error rate in the sampling determination table, to be in the range of 370–375 (Krejcie and Morgan, 1970). Based on this information, the research was conducted with 402 teachers. Table 1 shows the distribution of teachers participating in the research according to their demographic characteristics.

**TABLE 1:** Data on demographic characteristics of the participants.

Demographic variable	Groups	Frequency (*n*)	Percentage (%)
Gender	Female	224	56
	Male	178	44
	Total	402	100
Professional seniority	0–5 years	115	29
	6–10 years	81	20
	11–15 years	82	20
	16–20 years	75	19
	21 years or more	49	12
	Total	402	100
Graduation	Undergraduate degree	340	85
	Graduate degree	62	15
	Total	402	100
Level of education to work	Primary school	155	39
	Secondary school	147	36
	High school	100	25
	Total	402	100

According to Table 1, 224 of the 402 teachers were female (56%) and 178 were male (44%). In addition, 115 (29%) of the teachers have 0–5 years seniority, 81 (20%) 6–10 years of seniority, 82 (20%) 11–15 years of seniority, 75 (19%) 16–20 years of seniority, and 49 (12%) have 21 years or more of professional seniority. It was observed that 340 (85%) of the teachers were at the undergraduate level and 62 (15%) were at the graduate level. When the educational levels of the teachers were examined, it was observed that 155 (39%) teachers worked in primary schools, 147 (36%) teachers in secondary schools, and 100 (25%) teachers in high schools.

### Data Collection Process and Tools

In the research; Sustainable Leadership Scale developed by Çayak and Çetin (2018); Diversity Management Scale developed by Balay and Sağlam (2004), and Emotional Intelligence Scale developed by Titrek (2005) were used. The psychometric properties of data collection tools are presented below.

#### Sustainable Leadership Scale

The Sustainable Leadership Scale, which was developed by Çayak and Çetin (2018) and aims to measure the sustainable leadership behavior levels of school principals according to a teacher perception, is a five-point Likert type scale consisting of 4 sub-dimensions and 36 items. Thirty-six items with factor loadings between 0.55 and 0.79 explain 66.71% of the total variance. The goodness of fit values obtained as a result of the confirmatory factor analysis regarding the four-factor structure of the scale are as follows: x^2^/df = 3.55; TLI = 0.91; CFI = 0.92, and RMSEA = 0.06. The Cronbach’s Alpha internal consistency coefficients calculated for the sub-dimensions and it was found to be 0.97 for the Administrative Sustainability sub-dimension; 0.92 for the Economic Sustainability sub-dimension; 0.85 for the Cultural Sustainability sub-dimension; 0.87 for the Social Sustainability sub-dimension; and 0.98 for the overall scale.

#### Diversity Management Scale

The Diversity Management Scale, developed by Balay and Sağlam (2004), is a five-point Likert-type scale consisting of three sub-dimensions and 28 items. The variance rate explained by the sub-dimensions of the diversity management scale was 11.4% for the first sub-dimension, “Individual Attitudes and Behaviors”; 15.7% for the second sub-dimension, “Organizational Values and Norms”; and 31% for the third sub-dimension, “Managerial Practices and Policies.” The variance value of the Diversity Management Scale explained as a single factor is 41.7%. The Cronbach’s Alpha reliability coefficient of the scale was calculated as 0.77 for the first factor, 0.83 for the second factor and 0.95 for the third factor. In the study, the general reliability of the scale was calculated as 0.85. It can be said that as the scores obtained from the dimensions of the scale increase, teachers’ perceptions of the diversity management related to that dimension increase in a positive way.

#### Emotional Intelligence Scale

Donaldson-Feilder and Bond (2004) state that the use of self-filled (based on self-assessment) scales in the evaluation process of emotional intelligence can be potentially misleading. For this reason, it is important that the results of the studies on school administrators are more objective and that the scales are applied to other people who observe them, not to the people to be evaluated, in order to obtain more reliable results. Based on this information, the Emotional Intelligence Scale developed by Titrek (2005) was used in the research. The emotional intelligence scale is a five-point Likert-type scale consisting of five sub-dimensions and 72 items. The rate of variance explained by the sub-dimensions of the Emotional Intelligence Scale was 52% for the first sub-dimension “Self-Awareness (Awareness of Your Emotions),” 56% for the second sub-dimension “Regulating and Managing Emotions,” and 70% for the third sub-dimension, “Motivation of Emotions,” 52% for the fourth sub-dimension “Empathy” and 62% for the fifth sub-dimension “Social Skills.” The Cronbach’s Alpha reliability coefficient of the scale was 0.77 for the “Self-Awareness” sub-dimension, 0.81 for the “Regulating and Managing Emotions” sub-dimension, 0.83 for the “Motivation of Emotions” sub-dimension, 0.74 for the “Empathy” sub-dimension and 0.89 for the “Social Skills” sub-dimension. In the study, the general reliability of the scale was calculated as 0.96.

#### Personal Information Form

In the personal information form prepared by the researchers, questions about the gender of the participants, their professional seniority, their educational status, and their educational levels were included.

### Procedures and Data Analysis

The scale links prepared through Google forms were sent to the teachers who wanted to participate in the research voluntarily by the school administration. Data collected from 402 teachers were analyzed using the SPSS 22.0 program. In the analysis of the data obtained within the scope of the research, descriptive statistics related to the variables were calculated and the relations between the variables were determined using the Pearson product-moment correlation coefficient. Afterward, mediation analyses were made using the structural equation model and path analysis in line with the model proposed by Baron and Kenny (1986). Statistical programs (SPSS and AMOS) were used in the analysis of the data collected within the scope of the research. Before starting the analysis firstly, whether or not the data set had unidirectional and multidirectional normality assumption was analyzed. For this purpose, the skewness and kurtosis values of the data set and the Q-Q graphs were examined. Administrative sustainability (−0.01 to −0.08), economic sustainability (−0.21 to 0.09), cultural sustainability (0.31 to −0.10), social sustainability (0.44 to 0.07), sustainable leadership overall average (−0.01 to −0.24), individual attitudes and behaviors (0.14 to −0.40), organizational values and norms (−0.26 to 0.29), managerial practices and policies (−0.16 to 0.06), diversity management overall average (−0.08 to 0.15), self-awareness (0.11 to −0.15), managing emotions (−0.25 to −0.50), emotional motivation (−0.28 to 0.51), empathy (−0.10 to 0.14), social skills (−0.11 to 0.14), and emotional intelligence overall average (0.10 to 0.06) scores were within the normal distribution limits. George and Mallery (2003) state that when the skewness and kurtosis coefficients are in the range of ±2, the data show normal distribution. In addition, it has been observed that the expected and actual values of the data are distributed close to a line with a slope of 45 degrees in the created Q-Q charts. Therefore, this showed that the distribution of the data would be considered normal (Can, 2014). Since multivariate analyses were used in the study, it was also examined whether there was a multicollinearity problem between the variables. In this, the correlation values between the variables were examined. The correlation between the predictor variables above 0.80 indicates that there may be a multicollinearity problem, while the correlation above 0.90 indicates that there may be an important multicollinearity problem (Büyüköztürk, 2011). Based on this information, as seen in Table 2, there is no multicollinearity problem between sub-dimensions. In the analyses, the significance of the difference between the means was tested at the 0.05 level. In the interpretation of arithmetic averages, the range of 1.00–1.79 was evaluated as “very low,” the range of 1.80–2.59 as “low,” the range of 2.60–3.39 as “medium,” the range of 3.40–4.19 as “high,” and the range of 4.20–5.00 as “very high.” In addition, in the interpretation of the correlation analysis, the range of 0.00–0.30 was accepted as “low,” the range of 0.31–0.70 as “medium,” and the range of 0.71–1.00 as “high” level (Büyüköztürk, 2011). The study’s first question was investigated by the arithmetic mean, the second question by Pearson product-moment correlation analysis, the third question by *t*-test and one-way analysis of variance (ANOVA), and the fourth question by structural equation modeling (SEM).

**TABLE 2:** Descriptive statistics and correlation analysis of variables.

	1	2	3
1. Sustainable Leadership	1		
2. Emotional Intelligence	0.68*	1	
3. Diversity Management	0.50*	0.78*	1
Mean	3.90	3.95	3.87
Standard Deviation	0.32	0.33	0.36
Skewness	−0.01	0.10	0.00
Kurtosis	−0.24	0.06	−0.15

*p < 0.01.

## Findings

In the study, school principals’ sustainable leadership behaviors, their ability to manage differences and their emotional intelligence levels were examined and it was examined whether these showed significant differences according to some demographic variables. In addition, the results of the correlation analysis between the variables were given, and then the structural equation model regarding the mediating role of emotional intelligence levels in the relationship between school principals’ sustainable leadership behaviors and their ability to manage differences was tested.

The relationships between the arithmetic mean, standard deviation and skewness-kurtosis values of the scores obtained from the scales used in the research and the scale scores are presented in Table 2.

When the descriptive statistics in Table 2 are examined, it is seen that the teachers’ perceptions of sustainable leadership ($\bar{\text{X}}$ = 3.90), their ability to manage differences ($\bar{\text{X}}$ = 3.87) and their emotional intelligence perception levels ($\bar{\text{X}}$ = 3.95) are relatively high. In addition, when the skewness and kurtosis values of the variables discussed in the study are examined, it is seen that the distribution exhibits a normal distribution. Looking at the correlation coefficients between the variables in Table 1, there is a positive, moderately significant relationship between sustainable leadership and diversity management (*r* = 0.50; *p* < 0.001). Likewise, there is a positive, moderately significant relationship between sustainable leadership and emotional intelligence (*r* = 0.68; *p* < 0.001). In addition, a positive and highly significant relationship was found between emotional intelligence and diversity management (*r* = 0.78; *p* < 0.001).

An independent group *t*-test was conducted to determine whether the sustainable leadership scale, emotional intelligence scale and diversity management scale scores of the teachers constituting the sample group showed a significant difference according to the gender variable.

As can be seen in Table 3, as a result of the independent groups *t*-test, sustainable leadership (*t* = −0.34; *p* > 0.05), emotional intelligence (*t* = −1.60; *p* > 0.05), and diversity management (*t* = −0.59; *p* > 0.05) scores, the difference between the arithmetic means of the groups was not significant.

**TABLE 3:** Independent group *t*-test results conducted to determine whether sustainable leadership scale, emotional intelligence scale and diversity management scale scores differ according to teachers’ gender variable.

Scale	Groups	*N*	$\bar{\text{x}}$	Sd	Se	*t*-test		
						*t*	Df	*p*
Sustainable leadership	Female	224	3.89	0.32	0.02	−0.34	400	0.736
	Male	178	3.90	0.32	0.02			
Emotional intelligence	Female	224	3.92	0.33	0.02	−1.60	400	0.111
	Male	178	3.98	0.33	0.03			
Diversity management	Female	224	3.86	0.37	0.03	−0.59	400	0.559
	Male	178	3.88	0.34	0.03			

A one-way ANOVA was conducted in order to determine whether the scores of the sustainable leadership scale, the emotional intelligence scale and the diversity management scale of the teachers constituting the sample group show a significant difference according to the professional seniority variable.

As seen in Table 4, as a result of the one-way ANOVA, the difference between the arithmetic averages of the groups for the sustainable leadership score (*F* = 1.708; *p* > 0.05) according to the variable of professional seniority was not found significant. However, the difference between the arithmetic mean scores of the groups for emotional intelligence (*F* = 2.859; *p* < 0.05) and diversity management (*F* = 4.749; *p* < 0.05) scores according to the variable of professional seniority was found to be significant. Complementary analyses (*post-hoc*) were carried out in order to determine from which groups the significant difference detected for emotional intelligence and the diversity management originates. For this purpose, firstly, the homogeneity of variance was checked with Levene’s analysis and it was found that the variances were not homogeneous (L_*F*_ = 5.626; *p* < 0.05/L_*F*_ = 3.107; *p* < 0.05). For this reason, the Dunnet *C*-test was preferred. As a result of the Dunnet *C*-test, it was found that a significant difference for the emotional intelligence scale was in favor of teachers with 6–10 years of seniority between teachers with 6–10 years of seniority and those with 0–5 years of seniority. On the other hand, for the diversity management scale, the significant difference was found between teachers with 6–10 years of seniority and teachers with 0–5 years of experience, and between teachers with 16–20 years of seniority and teachers with 0–5 years of experience.

**TABLE 4:** Results of one-way analysis of variance (ANOVA) conducted to determine whether sustainable leadership scale, emotional intelligence scale and diversity management scale scores differ according to teachers’ professional seniority.

Scale	Groups	*n*	$\bar{\text{x}}$	Sd	Source of variance	Sum of squares	Df	Mean square	*F*	*p*	Dunnet C
Sustainable leadership	0–5 years (1)	115	3.84	0.35	Between groups	0.683	4	0.171	1.708	0.147	
	6–10 years (2)	81	3.90	0.28	within groups	39.674	397	0.100			
	11–15 years (3)	82	3.90	0.32	total	40.356	361				
	16–20 years (4)	75	3.95	0.32							
	Total	49	3.93	0.27							
Emotional intelligence	0–5 years (1)	115	3.86	0.36	Between groups	1.247	4	0.312	2.859	0.023	2-1
	6–10 years (2)	81	3.99	0.28	within groups	43.284	397	0.109			
	11–15 years (3)	82	3.95	0.34	total	44.530	401				
	16–20 years (4)	75	4.00	0.38							
	Total	49	3.98	0.21							
Diversity management	0–5 years (1)	115	3.95	0.33	Between groups	2.310	4	0.577	4.749	0.001	2-1 4-1
	6–10 years (2)	81	3.76	0.39	within groups	48.266	397	0.122			
	11–15 years (3)	82	3.91	0.31	total	50.576	401				
	16–20 years (4)	75	3.89	0.38							
	Total	49	3.98	0.31							

An independent group *t*-test was conducted to determine whether the sustainable leadership scale, emotional intelligence and diversity management scale scores of the teachers constituting the sample group showed a significant difference according to the variable of educational status.

As can be seen in Table 5, as a result of the independent groups *t*-test, the difference between the arithmetic averages of the groups for the sustainable leadership (*t* = −1.95; *p* > 0.05) score according to the educational status variable was not found significant. However, the difference between the arithmetic means of the groups for emotional intelligence (*t* = −2.54; *p* < 0.05) and diversity management (*t* = −2.43; *p* < 0.05) scores was found to be significant. The averages of the teachers at the graduate education level were found to be significantly higher than the averages of the teachers with the undergraduate education level.

**TABLE 5:** Independent group *t*-test results conducted to determine whether sustainable leadership scale, emotional intelligence scale, and diversity management scale scores differ according to the variable of educational status.

Scale	Groups	*N*	$\bar{\text{x}}$	Sd	Se	*t*-test		
						*t*	Df	*p*
Sustainable leadership	Undergraduate degree	340	3.88	0.32	0.02	−1.95	400	0.052
	Graduate degree	62	3.97	0.29	0.04			
Emotional intelligence	Undergraduate degree	340	3.93	0.32	0.02	−2.54	400	0.012
	Graduate degree	62	4.04	0.38	0.05			
Diversity management	Undergraduate degree	340	3.85	0.34	0.02	−2.43	400	0.016
	Graduate degree	62	3.97	0.40	0.05			

The mediating effect of emotional intelligence on the relationship between school principals’ sustainable leadership behaviors and diversity management skills was examined in line with the model proposed by Baron and Kenny (1986). According to this model, in order to test the mediator variable model, there must be a significant relationship between dependent, independent and mediating variables. When the findings in Table 2 were examined, it was seen that all variables had significant relationships among themselves. In addition, if the variable in which the mediation effect is investigated in a significant relationship between variables is added to the model, there is a decrease in the level of the relationship between the variables, this indicates the mediation effect. When the variable that is thought to play a mediating role is added to the model, the relationship between the dependent and independent variable is considered “full mediation”; If the relationship is significant and the effect level decreases, it is considered as “partial mediation.” In this study, it was investigated whether emotional intelligence has a mediating role in the relationship between sustainable leadership (independent variable) and diversity management (dependent variable).

**FIGURE 1:**
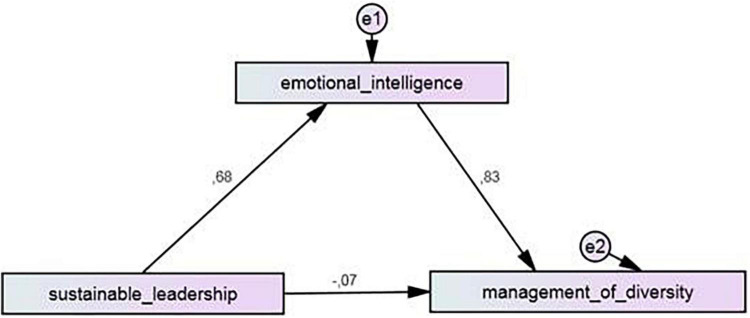
Path analysis diagram for prediction of diversity management (mediation effect).

The relationships between sustainable leadership, diversity management and emotional intelligence, which is considered as a mediating variable, were examined by path analysis. The results regarding the mediating effect of emotional intelligence between sustainable leadership and diversity management are shown in Figure 1 and Table 6.

**TABLE 6:** Findings on the mediating effect of emotional intelligence on the relationship between sustainable leadership and diversity management.

Predictor (Exogenous)	Predicted (Endogenous)	*B*	Se	*t*	β	*p*
***Direct effects***						
Sustainable Leadership →	Emotional Intelligence	0.72	0.04	18.76	0.68	0.000
Emotional Intelligence →	Diversity Management	0.89	0.05	19.57	0.83	0.000
Sustainable Leadership →	Diversity Management	0.55	0.05	11.41	0.50	0.000
***Indirect effects***						
Sustainable Leadership →	Diversity Management	−0.08	0.05	−1.74	−0.07	0.083

As can be seen in Table 6, sustainable leadership significantly positively predicts emotional intelligence (β = 0.68, *t* = 18.76, *p* < 0.001) and diversity management (β = 0.50, *t* = 11.41, *p* < 0.000). In addition, emotional intelligence significantly positively predicts the diversity management (β = 0.83, *t* = 19.57, *p* < 0.001). Kline (2015) suggested critical values for the evaluation of standardized effect sizes. If it is less than 0.10, it is low-level impact, if it is around 0.30, it is medium-level impact, and 0.50 and above is high-level impact. Accordingly, it can be said that sustainable leadership and emotional intelligence have a high effect on the diversity management. Sustainable leadership and emotional intelligence together were found to predict 61% of the variance in diversity management (*R*^2^ = 0.61, *p* = 0.000). The significance of the regression values between the variables was interpreted as the assumptions of the mediation test were realized. After adding emotional intelligence to the model as a mediator variable, the relationship between sustainable leadership and diversity management was found to be insignificant (β = −0.07, *t* = −1.74, *p* > 0.05). This finding indicates that emotional intelligence plays a full mediating role in the relationship between sustainable leadership and diversity management.

## Results and Discussion

In this study, sustainable leadership behaviors, diversity management skills and emotional intelligence levels of school principals were examined in line with the views of 402 teachers working in public schools in Pendik and Kartal districts of Istanbul in the 2020–2021 academic year. In addition, the mediating role of emotional intelligence in the relationship between the variables and the relationship between sustainable leadership and diversity management were examined.

Research findings showed that teachers’ perceptions of school principals regarding sustainable leadership behaviors are at a high level. In similar research results examining sustainable leadership in educational organizations (Yollu, 2017; Stephens, 2019; Dağdeviren-Ertaş, 2020), it was found that school principals exhibit high levels of sustainable leadership behaviors. In his study, Cook (2014), in which he examined how sustainable school leadership is perceived by teachers, participants stated that sustainable leadership is needed for students to continue their academic development and teachers’ professional development, and for this reason, sustainable leadership is important. In this context, it can be considered as a positive situation that school principals exhibit high levels of sustainable leadership behaviors in the findings obtained from both this research and other studies. The development of sustainable characteristics of school leaders is a factor that increases the success of the educational organizations they manage (Lambert, 2012).

Another finding of the study showed that teachers’ perceptions of school principals’ ability to manage diversity is at a high level. This finding of the study is similar to the results of some studies. E.g., as a result of their researches Memduhoğlu (2007), Young et al. (2010) and Özan and Polat (2013). determined that the perceptions of school principals on the management of diversities are at a high level according to teacher perceptions. However, in their research on educational organizations and Balyer and Gündüz (2010) and determined that teachers’ and administrators’ perceptions of the management of diversity are moderate. When the literature is examined, it is seen that the perceptions of the education administrators regarding the management of diversity are at a medium and higher level. It is a positive situation that the ability to manage diversities, which is an important point for a leader, is not low. It can be thought that the selection of school principals among senior and successful teachers was effective in achieving such a result. As a matter of fact, it can be thought that the ability to manage diversities can develop with the development of social intelligence of individuals with experience and work and life experience.

The findings showed that teachers’ perceptions of school principals regarding their emotional intelligence levels were high. Similar studies on educational organizations (Grobler et al., 2017; Erkoç, 2019) revealed that educators have high emotional intelligence levels. It is of great importance that school administrators, who are teaching leaders, also have emotional intelligence competencies (Recepoğlu, 2012). Because, studies reveal that school principals’ emotional intelligence levels are closely related to school success (Bardach, 2008).

Another finding of the study revealed that teachers’ perceptions of school principals’ sustainable leadership behaviors, diversity management skills and emotional intelligence levels did not differ significantly according to teachers’ genders. In their similar studies, Yollu (2017) and Dağdeviren-Ertaş (2020) found that school principals’ sustainable leadership behavior levels did not show a significant difference according to the gender variable according to teacher perceptions, while Çayak (2018) found that school principals’ sustainable leadership behavior levels were significantly different according to the gender variable of teachers. In this research, the scale scores of male teachers were found to be significantly higher than the scores of female teachers. The issue of gender is one of the basic concepts of diversity management philosophy. Thus; like the glass ceiling theory, one of the biggest obstacles women face in promotions in business life is gender differences. Especially with the increasing participation of women in active business life, companies that aim to turn this into a competitive advantage are trying to turn gender differences into wealth rather than a problem (Köksalan, 2019). In this context, it can be considered as a positive situation that teacher perceptions of school principals’ skill levels in managing diversity did not show a significant difference according to teachers’ genders. When the studies conducted in the context of educational organizations are examined, there are studies that support this finding of the research. E.g., Ekşi et al. (2016) and Küçük (2018) we are revealed that teachers’ perceptions of diversity management did not show a significant difference according to the gender variable. However, Balyer and Gündüz (2010) and Kılıçlar-Şahin (2015) revealed in their research that there is a significant difference in favor of male teachers in terms of gender in teachers’ perceptions of diversity management. On the other hand, Karademir et al. (2012) revealed that there is a significant difference in favor of female teachers in their research. Therefore, it can be said that the effect of the gender variable on the management of diversity may vary according to different factors such as the organizational structures of the researches or the participants of the research. Turanlı (2007) and Erkoç (2019) in similar studies in which they examined the emotional intelligence levels of school principals also found that there was no significant difference between the opinions of the participants according to the gender variable. This situation supports this finding of the research. However, it is possible to come across studies in the literature showing that there are significant differences between the opinions of the participants according to the gender variable. For example, Güçlü and Recepoğlu (2009) and Recepoğlu (2012) revealed that there are significant differences in favor of female teachers between the opinions of the participants according to the gender variable.

Findings of the study revealed that teachers’ perceptions of school principals’ sustainable leadership behaviors do not show a significant difference according to teachers’ professional seniority; however, it has been revealed that the perceptions of the diversity management skills and emotional intelligence levels show a significant difference according to the professional seniority of the teachers. For emotional intelligence, this difference is in favor of those with 6–10 years of seniority between teachers with 6–10 years of seniority and those with 0–5 years of seniority. For the diversity management, it is in favor of those with 6–10 years and 16–20 years of seniority between teachers with 6–10 years and 16–20 years of seniority and those with 0–5 years of seniority. In his study, Dağdeviren-Ertaş (2020) found that teachers’ perceptions of school principals’ sustainable leadership behaviors did not differ significantly according to professional seniority. However, in a similar study, Çayak (2018) revealed that school principals’ sustainable leadership behaviors showed a significant difference according to the variable of educational status. In this research, teachers with a seniority of 21 years or more perceive school principals as more sustainable than those with other seniority years.

As in other fields, employees in educational organizations have different demographic, social, cultural, and individual characteristics and it is important to manage them well in line with individual and organizational goals (Memduhoğlu, 2011). In this respect, it can be considered as an expected situation that the perceptions of school principals regarding the ability to manage diversities, as a characteristic of the career stages in which teachers with different professional seniority are in differ. Ekşi et al. (2016) found in a similar study that teachers’ perceptions of diversity management differed significantly according to professional seniority. In their similar studies, Yollu (2017) and Dağdeviren-Ertaş (2020) found that school principals’ sustainable leadership behavior levels did not show a significant difference according to the gender variable according to teacher perceptions, while Çayak (2018) found that school principals’ sustainable leadership behavior levels were significantly different according to the gender variable of teachers., they concluded that as the professional experience of the teachers increased, there was a positive change in the perceptions of the school administration regarding their ability to manage differences. However, unlike the result of this research, Balyer and Gündüz (2010) and Kılıçlar-Şahin (2015) found that the opinions of the participants on the management of diversity did not differ significantly according to professional seniority, as a result of their research in which they examined the perceptions of teachers about the management of diversity in schools. On the other hand, it was seen that different results were obtained in similar studies in which the emotional intelligence levels of the employees in educational organizations were examined according to the variable of professional seniority. In some of these studies, it was observed that the emotional intelligence levels of educators did not differ significantly according to the variable of professional seniority (Erkoç, 2019; Ertuğrul, 2020), while in some of them, it was observed that the levels of emotional intelligence differed significantly according to the variable of professional seniority (Çiçen, 2021).

Findings of the study revealed that teachers’ perceptions of school principals’ sustainable leadership behaviors, diversity management skills and emotional intelligence levels do not differ significantly according to a teachers’ educational status. There are few studies in the literature examining the perceptions of school principals regarding sustainable leadership behaviors according to a teachers’ educational status. Of these, Yollu (2017) and Çayak (2018) found that the factors according to the educational status variable in their research; found that school principals’ perceptions of sustainable leadership behaviors did not show a significant difference. However, there are studies conducted with different types of leadership using the educational status variable. For example, Değirmenci (2006) and Uygur (2010) found that a teachers’ educational background did not cause a significant difference between their perceptions of high school administrators’ levels of realizing their cultural leadership roles.

Karademir et al. (2012) and Özan and Polat (2013) who examined the perceptions of teachers regarding the management of diversity in educational organizations, found that teachers’ perceptions of diversity management did not show a significant difference according to the variable of educational status. The results obtained from the studies are in line with the results of this study.

Although there are many studies in the literature that show that the emotional intelligence levels of education administrators do not differ significantly according to their education levels and support the result of this research (Acar, 2007; Babaoğlan, 2010), it is seen that the employees with high education level are compared to those with low education levels. It is also possible to come across studies showing that individuals have a higher level of emotional intelligence (Canbulat, 2007; Gürbüz and Yüksel, 2008). Therefore, it should not be overlooked that emotional intelligence skills are not the skills learned in the education curriculum, but the skills that the individual gains through his own efforts and experiences.

Another finding of the study is that there are positive and significant relationships between sustainable leadership, diversity management and emotional intelligence. In the literature, no study was found in which all three variables were used together. However, it is frequently emphasized in the literature that organizations should encourage differences rather than ignore them, value the workforce from different backgrounds, and try to improve their ability to manage them, in order to gain a sustainable competitive advantage in differentiated markets (McCann and Kohntopp, 2017; Rahman, 2019; Jankelová et al., 2020). Therefore, when we look at the issue from the point of view of educational organizations, having a strong sustainable leadership structure of the organization for a sustainable success will be the key to the successful management of the diversities within the institution. In addition, studies conducted on both educational organizations and other organizations in the literature reveal that managers’ emotional intelligence levels show positive relationships with leadership behaviors (Qian et al., 2017; Boyatzis, 2018; Lone and Lone, 2018). In this respect, these findings obtained from the research are also supported by the literature.

Research findings showed that emotional intelligence has a full mediator role in the effect of school principals’ sustainable leadership behaviors on their ability to manage differences. While workforce diversities in schools provide various organizational advantages, they can also lead to some inconveniences. Avoiding these drawbacks depends on seeing the diversities as a wealth and managing them well (Memduhoğlu, 2011). Organization managers have great responsibilities in this regard. Their leadership behaviors are of great importance in the effective management of diversities. However, as the research findings reveal, it is very effective for leaders to understand the individuals in front of them, to empathize with them and to provide a strong organizational communication, in short, to use their emotional intelligence effectively in this process. It is possible to find many studies in the literature that reveal the mediating role of emotional intelligence between similar and different variables (Sarwar et al., 2017; Foster et al., 2018; Yan et al., 2018). In this context, it is important to find emotional intelligence as a mediating variable in this study.

### Limitations and Implications for Further Research

This research has some limitations. The research was conducted in public schools in Istanbul, Turkey’s largest city. A similar research can be done with teachers working in rural schools or private schools where fewer teachers work, and the results can be compared. Self-report data collection tools were used to collect the data of the study. Therefore, the data obtained are limited to the answers given by the participants to the measurement tools and the scope of the measurement tools. Considering this limitation, it is thought that it would be beneficial to use different methods such as observation and interview in future studies.

### Conclusion and Suggestions

In the light of the results obtained from this research, some suggestions can be made for researchers and practitioners. Many factors are effective in organizational structures and managerial processes. Therefore, comprehensive researches can be conducted to determine the impact of these factors on the management of diversity, including different types of organizations. The moderator effects of variables such as education level, age, branch, gender, school region can be examined between teachers’ perceptions of school administrators’ leadership behaviors and their perceptions of the management of diversities. Quantitative research method was used in this study. For this reason, qualitative or mixed research methods can be used in similar studies to have more detailed information about the relationships of the variables.

In addition, some suggestions can be made for practitioners in line with the results obtained from this research. In order to manage diversities in the most effective way, school administrators should adopt a management approach in which all teachers can express their opinions and suggestions clearly, and the diversities of individuals are seen as a wealth. In-service training can be given to school principals so that they can use their emotional intelligence effectively and efficiently. In addition, qualified sustainable leadership trainings can be provided for school principals to internalize the basic philosophy of sustainability, and thus, sustainable leadership skills of education administrators can be developed at the point of creating a sustainable society.

## Data Availability Statement

The raw data supporting the conclusions of this article will be made available by the authors, without undue reservation.

## Ethics Statement

The studies involving human participants were reviewed and approved by the Istanbul Medeniyet University Ethics Committee with the decision number 2021/10-02. Only the volunteered participants participated in this study. Written informed consent from the participants’ legal guardian/next of kin was not required to participate in this study in accordance with the national legislation and the institutional requirements.

## Author Contributions

SÇ devised the research idea, developed the research model, wrote the method part, ran the analytic calculations, limitations, and implications, checked for the literature and discussion part, and arranged the last version of the manuscript. ME ran the data collecting process, wrote the introduction part, performed the results, discussion, recommendations, limitations, and controlled the other parts in terms of language and contextual check for the manuscript. Both authors contributed to the article and approved the submitted version.

## Conflict of Interest

The authors declare that the research was conducted in the absence of any commercial or financial relationships that could be construed as a potential conflict of interest.

## Publisher’s Note

All claims expressed in this article are solely those of the authors and do not necessarily represent those of their affiliated organizations, or those of the publisher, the editors and the reviewers. Any product that may be evaluated in this article, or claim that may be made by its manufacturer, is not guaranteed or endorsed by the publisher.
